# Evaluation of a pointwise microcirculation assessment method using liquid and multilayered tissue simulating phantoms

**DOI:** 10.1117/1.JBO.22.11.115004

**Published:** 2017-11-14

**Authors:** Ingemar Fredriksson, Rolf B. Saager, Anthony J. Durkin, Tomas Strömberg

**Affiliations:** a Linköping University, Department of Biomedical Engineering, Linköping, Sweden; b Perimed AB, Stockholm, Sweden; c University of California, Beckman Laser Institute and Medical Clinic, Irvine, California, United States; d University of California, Department of Biomedical Engineering, Irvine, California, United States

**Keywords:** diffuse reflectance spectroscopy, optical phantoms, multilayered tissue model, microcirculation, inverse Monte Carlo, sampling volume

## Abstract

A fiber-optic probe-based instrument, designed for assessment of parameters related to microcirculation, red blood cell tissue fraction ($f_{\mathrm{RBC}}$), oxygen saturation ($S_{O_{2}}$), and speed resolved perfusion, has been evaluated using state-of-the-art tissue phantoms. The probe integrates diffuse reflectance spectroscopy (DRS) at two source–detector separations and laser Doppler flowmetry, using an inverse Monte Carlo method for identifying the parameters of a multilayered tissue model. Here, we characterize the accuracy of the DRS aspect of the instrument using (1) liquid blood phantoms containing yeast and (2) epidermis-dermis mimicking solid-layered phantoms fabricated from polydimethylsiloxane, titanium oxide, hemoglobin, and coffee. The root-mean-square (RMS) deviations for $f_{\mathrm{RBC}}$ for the two liquid phantoms were 11% and 5.3%, respectively, and 11% for the solid phantoms with highest hemoglobin signatures. The RMS deviation for $S_{O_{2}}$ was 5.2% and 2.9%, respectively, for the liquid phantoms, and 2.9% for the solid phantoms. RMS deviation for the reduced scattering coefficient ($\mu_{s}^{\prime }$), for the solid phantoms was 15% (475 to 850 nm). For the liquid phantoms, the RMS deviation in average vessel diameter (D) was 1  μm. In conclusion, the skin microcirculation parameters $f_{\mathrm{RBC}}$ and $S_{O_{2}}$, as well as, $\mu_{s}^{\prime }$ and D are estimated with reasonable accuracy.

## 1 Introduction

The skin is a multilayer structure with the epidermis containing melanin, dermis containing blood with various oxygen saturations, and subdermal structures. A comprehensive evaluation of human skin microcirculation involves assessing the local blood flow in the different vessels in the upper 1 to 1.5 mm, from ascending arterioles and descending venules, the upper dermal vascular plexa, and the capillaries.^1^ Optical techniques for assessing the microcirculation involve the laser Doppler flowmetry (LDF) technique analyzing temporal laser speckle fluctuations when light being Doppler-shifted when scattered by red blood cells (RBCs) mixes with unshifted light. Diffuse reflectance spectroscopy (DRS) estimates the tissue fraction of RBCs and their oxygen saturation, by utilizing the characteristic absorption properties of different chromophores (e.g., oxy- and deoxyhemoglobin), for the decomposition of tissue bulk absorption from a measured diffuse reflectance spectrum. Light illumination can be steady-state,^2^ temporally,^3^ or spatially^4^ modulated.

We have developed an optical fiber probe-based technique integrating LDF and DRS in a multiparameter multilayer skin model based on Monte Carlo simulations of light transport including absorption, scattering, and Doppler shifts. The tissue model contains parameters of oxygen saturation ($S_{O_{2}}$), the fraction of blood in the two dermis layers ($f_{\mathrm{RBC}}$; upper 200  μm and a lower semi-infinite), the average vessel diameter, the speed of the RBCs in different speed regions (<1, 1 to 10, >10  mm/s), the wavelength-dependent reduced scattering coefficient ($\mu_{s}^{\prime }$), the melanin fraction, the epidermal thickness, and the average vessel diameter D. A multistep fitting algorithm searches for model parameters ensuring an optimal fit of measured and modeled DRS spectra in the visible and near-infrared wavelength range (475 to 850 nm) at two source–detector distances (0.4 and 1.2 mm) and the LDF spectra recorded at 0.8 mm from the source.^5^ The $f_{\mathrm{RBC}}$, $S_{O_{2}}$, and the speed resolved perfusion are output parameters with important clinical value,^6^ whereas the other parameters may contain clinically valuable information but are important for accurate assessment of the output parameters. The technique is implemented in the PeriFlux 6000 Enhanced Perfusion and Oxygen Saturation System (EPOS) (Perimed AB, Järfälla, Stockholm, Sweden).

The aim of this study was to evaluate the accuracy of the estimated parameters in the multilayered skin model, regarding foremost $S_{O_{2}}$ and $f_{\mathrm{RBC}}$, using liquid and two-layered optical tissue simulating phantoms. Intralipid and blood were used together with yeast to enable phantoms having oxygen saturation that could be systematically varied. Silicone phantoms were manufactured mimicking epidermis with thicknesses in the range 70 to 300  μm including coffee as absorber and titanium dioxide (${\mathrm{TiO}}_{2}$) as scatterer. Thick silicone dermal phantoms were manufactured using ${\mathrm{TiO}}_{2}$ as scatterer in concentrations relevant to skin and bovine hemoglobin to mimic the absorption properties of blood.

## 2 Materials and Methods

### 2.1 Phantoms

Two types of phantoms were used in this study: (1) a liquid phantom consisting of blood and intralipid having oxygen saturation that could be systematically varied by adding yeast and (2) two-layered silicone-based solid phantoms.

#### 2.1.1 Intralipid-blood-yeast phantoms

Liquid phantoms were made from 20% Intralipid (Fresenius Kabi AB, Uppsala, Sweden) adding phosphate-buffered saline to achieve a pH of 7.4, to a total volume of 1000 ml. Bovine RBCs (Sierra for Medical Science, Whittier, California) were prepared by centrifugation and washing with phosphate-buffered saline to produce packed RBCs. Two sets of phantoms were prepared where 16 and 8 ml of RBCs were added to each phantom, resulting in a fraction of RBCs of 1.6% and 0.8%, respectively. The phantom was placed in a heated bath with a magnetic stirrer, keeping the temperature close to 37°C. The phantom was temperature stabilized and oxygenated by stirring for 20 min before beginning data collection. The oxygen partial pressure, $pO_{2}$, was measured using a Clark type electrode (O2-ADPT device with a MI-730 O2 microelectrode; Microelectrodes Inc.). Small batches of dry yeast (7 g packets; Red Star active yeast, Red Star Yeast Co., Cedar Rapids), diluted in 3 ml water of ∼37°C, was added to the solution to gradually decrease hemoglobin oxygenation. In the first experiment, 1/2-1 package was added. In the second experiment, this was then decreased to 1/4-1/3 package, more frequently, in order to cause a smoother decrease in $pO_{2}$. The $pO_{2}$ was monitored by a voltmeter, calibrated to 160 mmHg for the initial voltmeter value and 0 mmHg as the minimal voltmeter value. The expected RBC oxygen saturation (%) was calculated from $pO_{2}$ using Eq. (1) in Severinghaus^7^

$$S_{O_{2}}=\frac{100}{\frac{23,400}{{\left(pO_{2}\right)}^{3}+150pO_{2}}+1}.$$ (1)

#### 2.1.2 Two-layered phantoms

Solid two-layered phantoms were created to mimic the layered structure of skin tissue. Two bottom layer phantoms were employed to mimic the dermal properties of skin at two distinct concentrations of hemoglobin. Nine top layer phantoms were fabricated to mimic epidermal properties having three distinct fractions of melanin (using coffee as a surrogate) at three thicknesses. All of these phantoms were fabricated at the Beckman Laser Institute, University of California, Irvine, and used polydimethylsiloxate (PDMS) (Kit P-4, Eager Plastics, Chicago) as the base medium, following the general methods previously published by these authors elsewhere.^8^^,^^9^ These methods were slightly modified to suit the needs of this particular investigation and are articulated below.

To mimic the spectral properties of melanin, freeze-dried coffee has been shown to be a reasonable surrogate.^9^ Concentrations of 0, 2, and 4 g of coffee dissolved in acetone (per 100 ml PDMS) were used to fabricate three batches of epidermal phantoms that mimic various fractions of melanin in the skin, approximately covering a range of skin types I to IV according to the Fitzpatrick scale.^10^ In this instance, 10 g of freeze-dried coffee was added to 20 ml of acetone in a disposable beaker. As the coffee crystals were dissolved by the acetone, it formed a thick liquid mass that settled to the bottom of the beaker. Extracting this coffee concentrate from the beaker using an irrigation syringe, the coffee was added directly to the raw PDMS by mass and mixed prior to the addition of the curing agent. These three concentrations of coffee are from now on referred to as “no coffee,” “low coffee,” and “high coffee” epidermis phantoms. Titanium oxide [titanium(IV) oxide, anatase, Sigma Aldrich, Saint Louis] (0.17 g per 100 ml PDMS) was used to provide scattering properties in all batches. The vigorous mixing required to homogeneously distribute the absorbing and scattering agents will also introduce air bubbles in the viscous PDMS medium. To that end, the mixture is placed in a Nalgene vacuum chamber for ∼15 to 20 min in order to remove the air bubbles.^8^^,^^9^ For each batch, three thicknesses, simulating the epidermis, were fabricated. Rather than using rectangular molds of varying thickness, as proposed previously, we used 100- and 60-mm-diameter petri dishes. Here, a discrete volume of the mixed and degassed PDMS liquid was extracted using a 3-ml irrigation syringe and placed into the center of the petri dish. Each dish was placed on a spin coater (SCK-200 Digital Spin Coater Kit, Intras Scientific, New Jersey) at low RPM (<120) until the PDMS was evenly distributed along the bottom of the dish. The volume of PDMS used in each case correlates with the desired thickness of the cured phantom, based on the diameter of the dish used. For this study, target thicknesses were 100, 200 and 300  μm, which translates to 0.79 ml (100 mm dish), 1.57 ml (100 mm dish), and 0.85 ml (60 mm dish), respectively. Since cured PDMS is electrostatic, additional amounts of the mixed PDMS was added to the edges of the dish to provide additional structure and support for the purpose of increasing mechanical integrity in these noncritical areas to enable handling.

Because PDMS is hydrophobic, it is exceptionally challenging to incorporate hemoglobin or find a suitable proxy that mimics its characteristic spectral absorption features across visible and near-infrared domains.^11^ We have developed a method to utilize freeze-dried bovine hemoglobin (Sigma Aldrich) in these PDMS phantoms. First, the freeze-dried hemoglobin is vigorously mixed with a common surfactant (triton X-100, Sigma-Aldrich) to dissolve/soften the larger flakes. It is then sonicated (Bransonic M1800, Branson Ultrasonics) for 90 to 120 min (repeated over the course of 3 days) to further reduce the size of these freeze-dried hemoglobin particles. This process breaks down the hemoglobin flakes into submicron-sized particles (verified using microscopy). Acting more like a pigment (particle) rather than a dye (solution), the hemoglobin remains fixed in its chemical structure, yet only minimally contributes to the scattering properties of the resulting phantom, as verified by integrating sphere measurements described in Sec. 2.2 and illustrated in the scattering spectra shown in Fig. 7(a). It is worth noting that this form of hemoglobin is not an ideal proxy, as the freeze-drying process results in additional contributions of hemoglobin breakdown products such as methemoglobin to the mixture (as shown in the resulting absorption properties in Fig. 6). For the two dermal phantoms, 4 and 8 ml of this hemoglobin slurry (∼1 and 2 g of hemoglobin, respectively) was added to 350-ml PDMS. These two dermis phantoms are from now on referred to as the “low” and “high” hemoglobin concentration dermis phantoms. 0.6 g of titanium oxide was added to each, to approximate the same scattering properties of the epidermal layers. Molded in 100×100  mm plastic cases, these phantoms were 35 mm thick. As the actual concentrations of hemoglobin are not precisely controlled in this particular protocol, three thin samples were poured from each batch, so their respective optical properties could be determined via an integrating sphere-based inverse adding-doubling method described in Sec. 2.2.

**Fig. 1:**
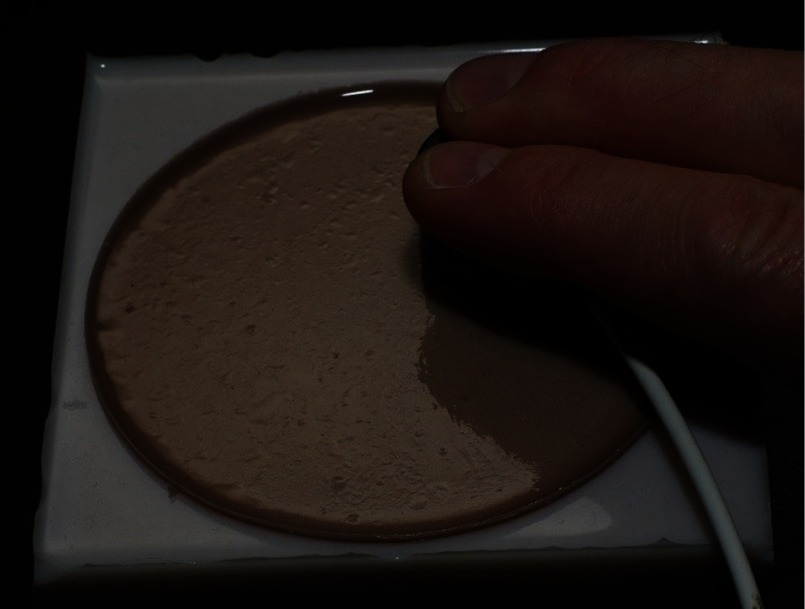
Measurement on a two-layered PDMS phantom. The thin epidermis-mimicking coffee phantom is placed on top of the thick dermis phantom, and the measurement probe is held in place by the fingers. Small air-bubbles and/or surface inhomogeneities can be observed in the two-layered phantom.

Measurements were performed on a total of 20 combinations of those epidermal and dermal phantoms. Either the dermis phantom with low or with high hemoglobin concentration was used in all combinations. On top of the dermal phantom either no epidermal phantom was used (2 cases), or an epidermal layer with any of the three coffee concentrations with any of the three thicknesses was used (18 cases). A metal rod was rolled over the thin phantom in order to reduce air bubbles so as to improve index of refraction matching between layers. The EPOS probe (see Sec. 2.3) was held in place by hand using a gentle pressure (Fig. 1). For each of the 20 phantom combinations, four separate measurements were performed positioning the probe halfway between the center and periphery of the epidermal layer on the two-layered phantoms and moved clockwise between the measurements.

### 2.2 Measurement of Phantom Optical Properties

In order to independently verify the respective optical properties of the two-layered phantoms, diffuse reflectance and transmittance spectra were measured for all thin samples (including thin samples concurrently fabricated from the two hemoglobin phantoms used in this investigation). These spectra were collected via a custom-built, validated single integrating sphere system specifically designed for broadband illumination and detection at the Beckman Laser Institute, a system that is thoroughly described in references.^9^^,^^12^^,^^13^ Each phantom set was measured at every thickness and at five spatial locations per sample to ensure homogeneity and consistency within each phantom set. Using the inverse adding-doubling code developed by Prahl,^14^^,^^15^ the absorption and reduced scattering coefficient spectra were calculated from the respective reflectance and transmittance measurements over a 450 to 1000 nm range at ∼1  nm resolution.

### 2.3 Measurement System

The microcirculation (phantom) parameters that were evaluated were measured using a PeriFlux 6000 EPOS system, integrating DRS and LDF in a fiber-optic probe.^5^ The system consisted of a PF6010 laser Doppler unit, a PF6060 spectroscopy unit, a broadband white light source (Avalight-HAL-S, Avantes BV, The Netherlands), and a fiber-optic thermostatic heating probe. The PF6060 unit had two spectrometers (AvaSpec-ULS2048L, Avantes BV) with optical notch filters mounted behind the 100-μm slit in order to suppress wavelengths 790±20  nm to ensure minimal influence from the PF6010 laser light on the DRS spectra. The fiber-optic probe consisted of two central emitting fibers and three detecting fibers. The fibers for the LDF laser light source and the detecting fiber at a distance of 0.8 mm had a diameter of 125  μm. Two detecting fibers were placed at a distance of 0.4 and 1.2 mm from the white light source fiber and were each connected to separate spectrometers. Those fibers had a diameter of 200  μm and all fibers had a numerical aperture of 0.37 and were made of fused silica. The probe was designed for measurements in reflection mode where in principle all detected light is scattered multiple times within the measured object. The data from the LDF unit were not used in this study.

An inverse Monte Carlo method is applied in the EPOS system in order to determine the parameters of a multilayered skin model. RBC oxygen saturation and tissue fraction as well as speed resolved perfusion are extracted from the skin model when the model is adapted to measured data. In this study, only DRS spectral data from the two spectrometers were used to determine the model parameters, hence no perfusion information was extracted. The inverse Monte Carlo method is thoroughly described elsewhere.^2^^,^^5^ In short, the forward problem of calculating DRS spectra at two source–detector separations for a given skin model is solved by applying Beer–Lamberts law on presimulated path-length distributions from each layer. The path-length distributions are presimulated for various epidermis thicknesses and scattering coefficients. The Beer–Lambert modified distributions from each layer are then merged together to give the detected intensity for a certain wavelength and source–detector separation. The inverse problem is a nonlinear optimization problem of fitting forward calculated spectra to the measured spectra while updating the model parameters in an iterative manner. The nonlinear optimization problem is solved using a trust region reflective algorithm with a tailor-made error function that for example emphasizes the spectral shape in the 500- to 600-nm hemoglobin absorption bands.

The model contained the 10 variable parameters $t_{\mathrm{epi}}$, α, β, γ, $c_{\mathrm{heme},1}$, $c_{\mathrm{heme},2}$, $S_{O_{2}}$, D, $f_{\mathrm{mel}}$, and $\gamma_{\mathrm{mel}}$. These are used to express the epidermis layer thickness and wavelength-dependent reduced scattering and absorption coefficients for all three layers according to: epidermis layer thickness ($t_{\mathrm{epi}}$); wavelength- (λ) dependent reduced scattering coefficient (same for all layers) expressed as

$$\mu_{s}^{\prime }(\lambda )=\alpha [(1-\gamma )\lambda^{-\beta }+\gamma \lambda^{-4}],$$ (2)

hemoglobin concentration (g/dl) in dermis layers ($c_{\mathrm{heme},1}$ and $c_{\mathrm{heme},2}$); hemoglobin oxygen saturation ($S_{O_{2}}$); average vessel diameter (D); melanin fraction in the epidermis layer ($f_{\mathrm{mel}}$); and melanin absorption shape ($\gamma_{\mathrm{mel}}$). The absorption spectrum of the epidermis layer was calculated according to

$$\mu_{a,\mathrm{epi}}(\lambda )=f_{\mathrm{mel}}6.6\times 10^{10}\lambda^{-\gamma_{\mathrm{mel}}},$$ (3)

whereas the absorption spectrum of the dermis layers was calculated according to

$$\mu_{a,n}(\lambda )=c_{\mathrm{heme},n}c_{\mathrm{vd}}(\lambda )\mu_{a,\mathrm{heme}}(\lambda ),$$ (4)

where $c_{\mathrm{vd}}$ is a compensation factor for the vessel packaging effect (note that $c_{\mathrm{vd}}\to 1$ when D→0)

$$c_{\mathrm{vd}}(\lambda )=\frac{1-\exp[D\mu_{a,\mathrm{heme}}(\lambda )]}{D\mu_{a,\mathrm{heme}}(\lambda )}$$ (5)

and

$$\mu_{a,\mathrm{heme}}(\lambda )=(1-S_{O_{2}})\mu_{a,\mathrm{Hb}}(\lambda )+S_{O_{2}}\mu_{{a,\mathrm{HbO}}_{2}}(\lambda ).$$ (6)

For the solid two-layered phantoms, $c_{\mathrm{heme},1}=c_{\mathrm{heme},2}$ and the vessel diameter D was set to zero, whereas $c_{\mathrm{heme},1}$ and $c_{\mathrm{heme},2}$ were allowed to differ and D was allowed to take any value for the liquid phantoms. The mean cell hemoglobin concentration was assumed to be 31  g/dl RBC for the bovine blood used in the liquid phantoms, thus giving the following relation between $f_{\mathrm{RBC}}$ and $c_{\mathrm{heme}}$

$$f_{\mathrm{RBC}}=c_{\mathrm{heme}}/0.31.$$ (7)

For the intralipid/blood-based liquid phantoms, $\mu_{a,\mathrm{Hb},\text{liquid}}$ was based on data from Ref. 16 and $\mu_{{a,\mathrm{HbO}}_{2},\text{liquid}}$ was based on data from Ref. 17. The reduced absorption spectrum ($\mu_{a,\mathrm{Hb},\mathrm{liquid}}$) was taken from Ref. 16, since it agrees better to data from other Refs. 18–20, whereas oxygenized absorption spectrum ($\mu_{{a,\mathrm{HbO}}_{2},\text{liquid}}$) was taken from Prahl, since it agrees better with data from the same references. In our experience, the spectral model generally fit *in vivo* measurements better (no systematic residual) when using those spectra than when choosing both spectra from either Refs. 16 or 17 (unpublished data), an observation that holds also for this study. For the solid two-layered phantoms, $\mu_{a,\mathrm{Hb},\text{solid}}$ was set to the measured absorption spectrum of any of the dermis phantoms, i.e., with low or high hemoglobin concentration, whereas the artificial saturated dermis phantom absorption spectrum $\mu_{{a,\mathrm{HbO}}_{2},\text{solid}}$ was set to

$$\mu_{{a,\mathrm{HbO}}_{2},\text{solid}}(\lambda )=\mu_{a,\mathrm{Hb},\text{solid}}(\lambda )\frac{\mu_{{a,\mathrm{HbO}}_{2},\text{liquid}}(\lambda )}{\mu_{a,\mathrm{Hb},\text{liquid}}(\lambda )}.$$ (8)

**Fig. 2:**
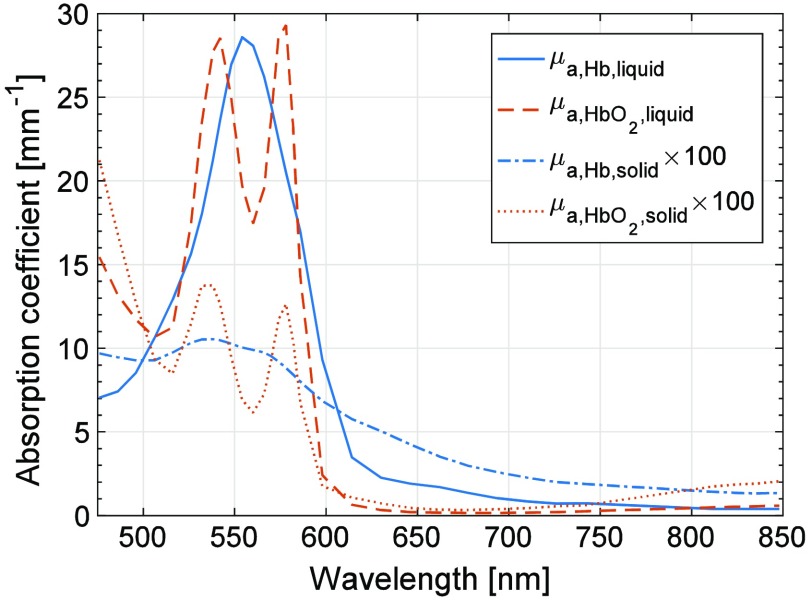
Comparison of the absorption spectra of hemoglobin with concentration relevant for normal blood and phantoms {high concentration hemoglobin phantom and its artificial “saturated” counterpart [Eq. (8)]}. The latter two have been rescaled in order to be more easily seen.

In that way, the relative difference between the oxygenized and reduced absorption spectra in the two-layered solid phantoms was the same as in the original model. When adapting the model to those phantom measurements, an oxygen saturation $S_{O_{2}}$ of zero is thus expected. The absorption spectra $\mu_{{a,\mathrm{HbO}}_{2},\text{liquid}}$ and $\mu_{a,\mathrm{Hb},\text{liquid}}$ as well as scaled absorption spectra of $\mu_{{a,\mathrm{HbO}}_{2},\mathrm{solid}}$ and $\mu_{a,\mathrm{Hb},\mathrm{solid}}$ for the high concentration hemoglobin phantom are found in Fig. 2.

### 2.4 Sampling Volume

In order to interpret the results obtained from measurements of the two-layered phantoms in a relevant manner, it is necessary to account for the fraction of the sampling volume that is included in each layer. The sampling volume for a specific model was defined as the average sampling volume over included wavelengths and over both source–detector separations. For each wavelength and source–detector separation, the sampling volume was estimated as previously described in Ref. 5. When considering, for example, the expected reduced scattering coefficient from the two-layered phantoms, the reduced scattering from the two layers was weighted according to the fraction of the sampling volume contained in each layer.

### 2.5 Data Exclusion

**Fig. 3:**
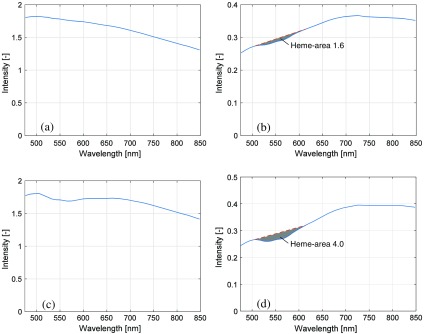
Example of DRS spectra at source–detector distance of (a and c) 0.4 mm and (b and d) 1.2 mm, with heme-areas marked in gray in (b) and (d). The spectra in (a) and (b) originate from one of the four measurements on the phantom with low hemoglobin dermis and thin epidermis with low coffee concentration, with a heme-area of 1.6. The spectra in (c) and (d) originate from one measurement on the phantom with high hemoglobin dermis and thin epidermis with no coffee phantom, with a heme-area of 4.0.

The analysis method in the EPOS system relies on the spectral shape of the diffuse reflectance spectra for determining the model parameters. For a robust determination of those parameters, distinct spectral characteristics, especially in the wavelength interval 500 to 600 nm (Fig. 2), where both reduced and oxygenated hemoglobin have characteristic absorption peaks, are a necessity. Therefore, data were excluded when the abovementioned wavelength interval contained too little characteristic hemoglobin-related spectral shape. This was done using an approach similar to what has previously been presented by Jonasson et al.^21^^,^^22^ for *in vivo* measurements. The exclusion criterion that was determined in those studies, and which is applied here, is that the magnitude of the area bounded by the measured spectrum and a line between the intensities at 506 and 614 nm had to be greater than 1.5 in order to be included in the study. The spectrum from the long source–detector separation (1.2 mm) was used when determining this exclusion criterion. Two examples of this area are shown in Fig. 3; one measurement with almost minimal acceptable heme-area [Figs. 3(a)–3(b); low coffee 75-μm epidermal layer on low hemoglobin dermal phantom, Table 1], and one with a higher heme-area [Figs. 3(c)–3(d); no coffee 90-μm epidermal layer on high hemoglobin dermal phantom, Table 1]. Note that the Hb optical signature is not visible in the short distance DRS spectrum in Fig. 3(a), whereas it is visible in Fig. 3(c). Based on the heme-area criterion, measurements from a total 11 of the 20 solid phantoms were included (see Table 1). All measurements from the liquid phantoms were included. As an example, measurements on forearm skin normally display a heme-area well above 1.5. The heme-area is normally lower than 1.5 for very low RBC tissue fractions (<0.1%).

**Table 1:** List of solid phantoms with epidermis thickness and average heme-areas for the four measurements of each phantom. Phantoms with average heme-area <1.5 were excluded.

Epidermis		Heme-area (–)	
Thickness (μm)		Low hemoglobin	High hemoglobin
None	—	**3.5**	**3.8**
No coffee	90	**3.5**	**4.0**
	155	**3.5**	**4.0**
	270	**3.5**	**3.6**
Low coffee	75	**1.5**	**2.6**
	160	0.7	1.5
	260	0.7	1.2
High coffee	70	1.3	**2.3**
	160	0.8	1.4
	210	0.7	1.2

## 3 Results

### 3.1 Liquid Phantoms

**Fig. 4:**
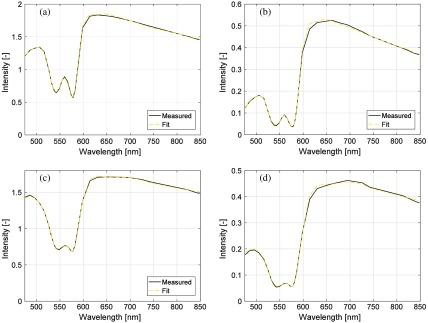
Examples of measured spectra and calculated spectra from best fit model at (a and b) 10 and 60 min in the first phantom, for source–detector separation (a and c) 0.4 mm and (b and d) 1.2 mm. At 10 min, the estimated $S_{O_{2}}=96\%$ compared to expected 99%, and estimated $f_{\mathrm{RBC}}=1.49\%$ compared to expected 1.60%. Corresponding numbers at 60 min were 47%/58% and 1.46%/1.60% for $S_{O_{2}}$ and $f_{\mathrm{RBC}}$, respectively.

Examples of the spectral fit at two points in time (10 and 60 min) in the first liquid phantom with $f_{\mathrm{RBC}}=1.6\%$ are shown in Fig. 4.

**Fig. 5:**
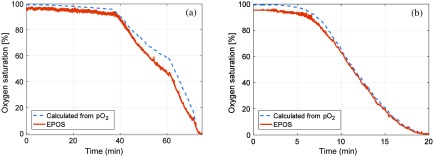
Estimated oxygen saturation from the EPOS system compared to calculated values based on measured ${\mathrm{pO}}_{2}$ for the two liquid phantoms with $f_{\mathrm{RBC}}$ of (a) 1.60% and (b) 0.80%.

The liquid phantom temperature was between 37.2°C and 37.7°C for the two experiments. The first experiment took 75 min to complete. A first low dose of yeast was added during the first 10 min. This caused a very small decrease in $S_{O_{2}}$. Higher doses of yeast (see Sec. 2.1) were added causing visible changes in $S_{O_{2}}$ at time points 38, 60, and 72 min [see Fig. 5(a)]. For the second experiment, taking 20 min, yeast was added in intermediate doses more frequently. In this experiment, the time points when adding the yeast were not observable.

Estimated oxygen saturation [$S_{O_{2}}$, Eq. (6)] over time for the two liquid phantoms is shown in Fig. 5 and compared with calculated $S_{O_{2}}$ values based on the measured $pO_{2}$ [Eq. (1)]. The root-mean-square (RMS) deviation from values calculated from the $pO_{2}$ readings was 5.2% units for the first measurement and 2.9% units for the second.

The estimated $f_{\mathrm{RBC}}$ was 1.49±0.03% (mean±standard deviation) compared to expected $f_{\mathrm{RBC}}=1.60\%$ for the first liquid phantom, with an RMS deviation for all samples of 11%. For the second phantom, corresponing numbers were 0.85±0.02%, expected 0.80%, RMS deviation 5.3%. The estimated average vessel diameter D was 1±1 and 2±2  μm, respectively, in the measurements of the two phantoms. For these homogeneous phantoms without blood vessels, the expected vessel diameter is 0  μm.

### 3.2 Two-Layered Phantoms

**Fig. 6:**
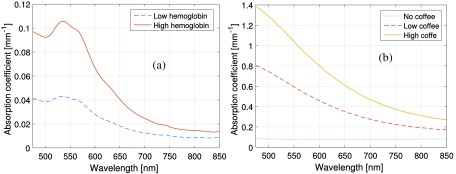
Absorption spectra of (a) the two hemoglobin phantoms and (b) the three epidermis phantoms, measured as described in Sec. 2.2.

**Fig. 7:**
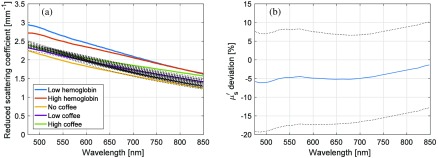
(a) Measured $\mu_{s}^{\prime }$ for the five types of phantoms (solid, thick) and estimated $\mu_{s}^{\prime }$ from the 44 included measurements on two one-layered and nine two-layered phantoms (dotted). (b) Estimated $\mu_{s}^{\prime }$-deviation from sampling volume weighted measured values as function of wavelength for the 44 included measurements. Average (solid) ±standard deviation (dotted).

Absorption spectra for the two hemoglobin phantoms as well as for the three concentrations of coffee in the epidermis phantoms are found in Fig. 6. The reduced scattering coefficients, $\mu_{s}^{\prime }$, of the phantoms are included in Fig. 7.

The optical and geometrical properties of the phantoms were used to create models representing the phantoms. From those models, the fraction of the sampling volume that was located in the hemoglobin layer was estimated. At least 75% of the sampling volume was located in the dermis layer—the fraction was the lowest for the hemoglobin phantom with higher absorption, higher for thinner epidermis layers, and higher for the epidermis phantoms with higher absorption.

The RMS deviation from zero in the estimated $S_{O_{2}}$ was 2.9%, where the maximum deviation in the 44 included measurements was 5.1%.

The estimated concentration of hemoglobin absorbers, $c_{\mathrm{heme}}$, in the sampling volume was generally overestimated. The relative RMS deviation for $c_{\mathrm{heme}}$ for the 44 included measurements (heme-area >1.5; four measurements on each of the 11 included phantoms) was 25%, whereas the relative RMS deviation for the four phantoms (16 measurements, all from the high hemoglobin phantom) with the most pronounced hemoglobin signature (heme-area >3.5) was 11%.

The RMS deviation of the estimated epidermis thickness was 137  μm for the 44 included measurements. The results for the estimated epidermis layer absorption showed a general high overestimation.

The relative RMS deviation of the estimated reduced scattering coefficient from the measured reduced scattering coefficient taking the sampling volume into account was 15% for the included wavelengths (475 to 850 nm). The spectral shape of the scattering was generally well estimated, as can be seen in Fig. 7(a). The average deviation in estimated $\mu_{s}^{\prime }$ was −4.6%, −6.0% for wavelengths <500  nm and −1.9% for wavelengths >800  nm.

## 4 Discussion

We used state-of-the-art liquid and two-layered solid optical phantoms in order to evaluate the accuracy of a system for comprehensive microcirculation assessment in a multilayer skin model. The fiber-optic system integrates DRS and LDF for assessing the output parameters $f_{\mathrm{RBC}}$, $S_{O_{2}}$, and speed resolved perfusion. These output parameters are influenced by the intrinsic skin model parameters such as epidermal thickness $t_{\mathrm{Epi}}$, reduced scattering coefficient $\mu_{s}^{\prime }$, and average vessel diameter D. It is, therefore, important to assess the accuracy of both intrinsic and output model parameters. The results show that the system is able to estimate the output parameters $f_{\mathrm{RBC}}$ and $S_{O_{2}}$ in an accurate manner (RMS deviation <15%). The accuracy is in line with what others have reported using similar systems on phantoms.^23^^–^^25^ The intrinsic skin model parameters $\mu_{s}^{\prime }$ and D are also estimated within 15%, whereas the error for the epidermal thickness and melanin fraction are much larger.

The Hb signature in the recorded DRS spectra is important for accurately assessing the blood-related parameters. This signature is most easily observed in the wavelength range 500 to 600 nm where Hb depicts clear absorption peaks, as seen in Fig. 2, distinctly different between oxygenized ($\mu_{{a,\mathrm{HbO}}_{2},\text{liquid}}$) and reduced ($\mu_{a,\mathrm{Hb},\mathrm{liquid}}$) states. We have defined a criterion based on this Hb signature, the heme-area, with an empirical cut-off value below 1.5 for excluding measurements. This value is based on previous studies in human skin.^21^^,^^22^ The heme-area for the liquid phantom experiments (13 and 16, respectively) show that the hemoglobin signature is clear in these spectra. It also shows that the heme-area is nonlinearly related to the $f_{\mathrm{RBC}}$. For these measurements, $f_{\mathrm{RBC}}$ was estimated with an RMS deviation of 11% at $f_{\mathrm{RBC}}=1.6\%$ and 5.3% at $f_{\mathrm{RBC}}=0.8\%$.

There is a difference in the inverse Monte Carlo method used here and the Monte Carlo-based approaches used by most others within the field of DRS. In other comparable methods,^3^^,^^4^^,^^26^^,^^27^ $\mu_{a}$ and $\mu_{s}^{\prime }$ are first estimated for each wavelength, and in a second step the amount of each chromophore is determined by linearly fitting the chromophores and their absorption spectra to the estimated total $\mu_{a}(\lambda )$. That approach requires a robust discrimination of $\mu_{a}$ and $\mu_{s}^{\prime }$ over the entire wavelength range, which may be challenging especially for wavelengths were values of $\mu_{a}$ are in the same range or higher than $\mu_{s}^{\prime }$. With the approach used in the PeriFlux 6000 EPOS system, all skin model parameters are fitted to measured intensity spectra simultaneously, where the spectral shapes of $\mu_{a}$ and $\mu_{s}^{\prime }$ are limited by the skin model parameters as given in Eqs. (1)–(5).

The inverse Monte Carlo method was designed foremost to estimate the RBC tissue fraction and oxygen saturation accurately. The included wavelengths as well as the nonlinear optimization strategy and the error function used by the optimization algorithms have been chosen and fine-tuned with that in mind.^5^ This emphasis in the algorithm becomes evident in this study, where the $S_{O_{2}}$ and $f_{\mathrm{RBC}}$ are well estimated. The average vessel diameter and the reduced scattering coefficient are also relatively well estimated, whereas the thickness and absorption properties of the epidermis layer are poorly estimated. Although the epidermis parameters are poorly estimated, we have previously shown that it is important to include those parameters as variable parameters in the model.^2^

The main purpose of the liquid phantoms was to assess the oxygen saturation, $S_{O_{2}}$, accuracy. Results obtained for oxygen saturation demonstrated an RMS deviation of 5.2% and 2.9%, respectively, for the two liquid phantoms used in the study when optically determined $S_{O_{2}}$ is compared to calculated values based on measured $pO_{2}$. The largest deviation was 10% at t=60  min in the first experiment [Fig. 5(a)]. The EPOS estimated $S_{O_{2}}$ changed more rapidly than predicted from $pO_{2}$, possibly due to a decrease in pH or increase in $p{\mathrm{CO}}_{2}$. This might be due to a higher development of ${\mathrm{CO}}_{2}$ during the reduction of the larger $f_{\mathrm{RBC}}$ in this experiment or to other factors changing pH. No such deviation was observed in the second experiment [Fig. 5(b)] at a lower $f_{\mathrm{RBC}}$. Our experiments were controlled for temperature, which was held within less than 1°C. The effects of changes in temperature, Ph, and $p{\mathrm{CO}}_{2}$ can be calculated according to Ref. 28. The EPOS data from the first experiment [Fig. 5(a)] agree well with that calculated from $pO_{2}$ for pH=7.2 (data not shown). In that case, the RMS deviation was 2.4% instead of 5.2% when pH of 7.4 was assumed.

Another method that could be applied to decrease hemoglobin oxygenation is by bubbling a gas such as pure $N_{2}$ through the phantom. This was done by Rejmstad et al.^29^ However, they used a very small phantom that was not suitable for the EPOS probe. In their study, they also pointed out the possibility of a change in pH during the experiment, despite using a phosphate-buffered solution. Furthermore, such an experiment could optimally be done by gradually lowering $pO_{2}$ by mixing pure $N_{2}$ and pure $O_{2}$ in different proportions, each at a stable level, rather than just feeding pure $N_{2}$ to the phantom. In summary, the experiment with the liquid phantom can be improved by further optimization of the procedure for altering the oxygen saturation.

Compared to fresh hemoglobin, the spectral characteristics of the absorption of the freeze-dried hemoglobin used in the solid phantoms are damped, as shown in Fig. 2. This damping in spectral features leads to a number of difficulties for the inverse Monte Carlo method employed in the EPOS system. The first of these is the exclusion of a number of our phantom measurements due to absence of spectral Hb signature in the 500- to 600-nm interval, as described in Sec. 2.4. With the spectral signature of normal hemoglobin (i.e., a combination of $\mu_{{a,\mathrm{HbO}}_{2},\text{liquid}}$ and $\mu_{a,\mathrm{Hb},\mathrm{liquid}}$), tissue with much higher epidermis absorption and with less hemoglobin than in the solid phantoms can be reliably analyzed. The risk of spectral cross-talk between hemoglobin and epidermis absorption is larger in the solid phantoms since the shape of the freeze-dried hemoglobin absorption spectrum differs less from the coffee absorption (see Fig. 6). In addition, the vessel packaging effect, which has been discussed in Ref. 30, becomes less distinct. Therefore, we chose not to investigate that effect within the context of the solid phantoms, as stated in Sec. 2.3. There is ongoing work to identify a hemoglobin-mimicking chromophore that can be included in a solid phantom with spectral features closer to that of fresh hemoglobin.

In the model, the spectral shape of the reduced scattering and epidermis absorption is limited by a few parameters according to Eqs. (2) and (3). The reduced scattering is also the same for both layers in the model. These model limitations prevent the model from exactly mimicking the solid phantoms and may account for some of the deviations in the estimated parameters.

In conclusion, we have used state-of-the-art skin tissue mimicking optical phantoms in order to evaluate the accuracy of the PeriFlux 6000 EPOS system. The results show that the system is able to estimate the output parameters RBC tissue fraction and oxygen saturation as well as the reduced scattering coefficient and the average vessel diameter with reasonable accuracy.
